# Supplementary material for: Development of a Responsible Policy Index to Improve Statutory and Self-Regulatory Policies that Protect Children’s Diet and Health in the America’s Region
Source: Int J Environ Res Public Health. 2020 Jan 13;17(2):495. doi: 10.3390/ijerph17020495 (PMC7013653; doi:10.3390/ijerph17020495)
Supplement: Supplementary file 1 [file ijerph-17-00495-s001.pdf]

Supplemental Table S1. Highest RESPI * scores for policies that restrict the marketing of HFSS ** food and beverage products to children in eight countries in the America's Region, 2010 to 2019

Country	Policy Document	Year ***	Policy Characteristics							Marketing Techniques Covered									RESPI * Score
			Policy type	Rights approach	Monitoring and evaluation	Sanctions	Nutrient criteria	Age of children	***Policy score***	Branding	Cartoon mascots, licensed and media characters, celebrities	Direct marketing	Point of sale	Premium offers	Product design and packaging	Product placement	Sponsorship	***Marketing score***	0 = lowest<br>10 = highest
Chile	Ley 20.606 Sobre la Composición de los Alimentos y su Publicidad	2018	1	0	0.5	0.5	1	0	1	1	1	1	1	1	1	1	1	5.0	8.0
Brazil	Resolução-RDC No- 24	2010	1	1	0	0.5	0.5	0	1	1	1	1	0	1	1	0	1	3.8	7.3
Canada	Guidelines for Food and Beverage Sales in B.C. Schools	2013	1	0	0.5	0.5	1	1	1	1	0	0	1	1	0	1	1	3.1	7.1
Uruguay	Rotulado de los alimentos envasados en ausencia del cliente, librados al consumo en el territorio nacional	2018	1	1	0.5	0	0.5	1	1	0	1	0	1	1	1	0	0	2.5	7.0
Ecuador	Reglamento Sanitario Sustitutivo de Etiquetado de Alimentos Procesados para el Consumo Humano	2014	1	1	0.5	0.5	1	1	1	0	1	0	1	0	1	0	0	1.9	6.9
Mexico	Proyecto de Modificación a la Norma Oficial Mexicana NOM-051-SCFI/SSA1-2010	2019	1	1	0.5	0	0.5	0	1	0	1	0	1	1	1	0	0	2.5	6.0
Brazil	Resolução 163 Conanda	2014	1	1	0.5	0.5	1	1	0	0	1	0	0	1	1	1	0	2.5	6.5
Uruguay	Ley no 19.140 Alimentación Saludable en los Centros de Enseñanza	2013	1	0	0.5	0.5	1	0	1	1	0	1	1	1	0	0	1	3.1	6.1

* RESPI: Responsible Policy Index (RESPI) to assess the strength of policies that restrict the marketing of HFSS food and beverage products to children. Highest RESPI scores = policies with a score of 6 and above. ** HFSS: high in fat, sugar and salt. *** Year: date of last amendment or modification to the policy. To calculate the RESPI scores three steps were followed: Step 1: Calculate the total points from the component 1 "policy characteristics". The total points are the sum of the points from each indicator of this component. Step 2: Calculate the total points from the component 2 "marketing techniques". [[total marketing techniques points] × 100/8]/20]. *Exceptions*: if the policy covers the following media channel, platform or setting: Broadcast and print: [[total marketing techniques points] × 100/6]/20; Outdoors and transportation: [[total marketing techniques points] × 100/4]/20. Step 3: Calculate the overall RESPI score for each policy on the marketing of HFSS food and beverage products to children. [total policy characteristics points] + [total marketing technique points].

Supplemental Table S2. Obesity prevalence for boys and girls aged 5–19 years in 14 countries of the Americas Region, 2016 and predicted by 2030.

Country	Obesity Boys 5–9 Years 2016 (%)	Obesity Girls 5–9 Years 2016 (%)	Obesity Predicted in Children 5–9 Years by 2030 (%)	Obesity Boys 10–19 Years 2016 (%)	Obesity Girls 10–19 Years 2016 (%)	Obesity Predicted 10–19 Years by 2030 (%)	Children with Obesity 5–19 Years Predicted by 2030
Argentina	25.6	17.8	27.2	18.3	10.4	20.0	1,262,281
Bolivia	12.7	10.8	18.3	8.2	7.3	13.6	600,564
Brazil	17.6	12.4	22.8	10.1	7.8	15.7	1,757,471
Canada	16.8	11.5	18.0	13.6	9.1	15.0	1,109,002
Chile	21.4	15.8	24.8	15.6	11.5	19.8	774,647
Colombia	9.8	7.4	14.4	6.3	6.0	11.1	1,583,123
Costa Rica	16.2	15.0	24.2	11.0	10.5	18.9	224,250
Dominican Republic	19.7	17.4	27.7	13.7	12.5	22.0	719,596
Ecuador	13.8	10.5	18.9	8.9	7.2	14.0	750,556
El Salvador	14.2	15.3	22.1	9.4	11.1	17.2	329,047
Mexico	19.9	15	23.6	15.2	11.7	19.3	6,550,276
Peru	12.3	8.9	16.1	7	5.8	10.9	1,079,543
Uruguay	20	14.4	22.4	14.2	10.0	17.2	133,317
USA	25.1	20.3	26.3	22.3	19.0	24.2	16,986,603

Source: World Obesity Federation. *Global Atlas on Childhood Obesity*. October 2019. https://www.worldobesity.org/nlsegmentation/global-atlas-on-childhood-obesity.
